# Aseptic Meningitis With an Isolated Positive Ocular Globe Compression Sign Diagnosed by Repeat Lumbar Puncture

**DOI:** 10.7759/cureus.32036

**Published:** 2022-11-30

**Authors:** Yuichiro Mine, Taiju Miyagami, Satoshi Furuya, Yusuke Kondo, Toshio Naito

**Affiliations:** 1 Clinical Training Center, Juntendo University Hospital, Tokyo, JPN; 2 Department of General Medicine, Juntendo University Hospital, Tokyo, JPN

**Keywords:** physical educatilon, viral meningitis, ocular globe compression, lumbar puncture (lp), meningitis pain

## Abstract

Aseptic meningitis is diagnosed using clinical and laboratory findings of meningeal inflammation in the absence of bacteria in cerebrospinal fluid smear and culture. It is commonly caused by a viral infection, and most cases are improved without specific treatment. We present a case of aseptic meningitis in a 33-year-old Japanese man that was diagnosed only after a repeat lumbar puncture. The patient had a positive ocular globe compression sign with no other positive meningeal signs. This case highlights the importance of repeated lumbar puncture in patients with suspected aseptic meningitis if the initial lumbar puncture results are negative, and there is a clinical value in assessing the ocular globe compression sign, particularly when other clinical signs of meningitis are absent.

## Introduction

Aseptic meningitis is diagnosed using clinical and laboratory findings of meningeal inflammation in the absence of bacteria in the cerebrospinal fluid (CSF) smear and culture. Aseptic meningitis can be classified into two broad etiological categories: infectious (including viral, bacterial, fungal, parasitic, or mycobacterial) or non-infectious (including drug-induced, neoplasm-related, systemic disease-related, or vaccine-related) [1]. It is commonly caused by viral infection, and most cases can be improved without specific treatment. Conversely, asymptomatic meningitis due to fungal, tuberculous, acute HIV infection, neurosyphilis, drugs, or carcinoma requires therapy [2].

Diagnostic clues for aseptic meningitis include positive meningeal sign tests such as nuchal rigidity, jolt accentuation test, Kernig and Brudzinski signs, and specific CSF findings such as elevated cell count and protein level as well as mildly decreased glucose level [1]. In this report, we present the case of a 33-year-old Japanese man with aseptic meningitis diagnosed only after repeating the lumbar puncture. The patient was suspected of having aseptic meningitis owing to positive ocular globe compression signs.

## Case presentation

A 33-year-old Japanese man presented to the emergency room with worsening headache, fever, and chills for the past day. He was not receiving any specific medication prior to his visit to our hospital. Neither he nor anyone else in his family had a history of tuberculosis. CSF and blood samples were collected for culture because of suspected meningitis and bacteremia. CSF was within normal limits (Table 1), and the blood culture was negative. The patient’s symptoms were treated with oral acetaminophen (3000 mg/day), and he was kept under observation in the outpatient clinic. He was hospitalized three days later for worsening headache and fever. On admission, he had a Glasgow Coma Scale of E4V5M6. His vital signs were as follows: body temperature, 38.4°C; pulse rate, 99 beats/min; blood pressure, 122/72 mmHg; and respiratory rate, 20 breaths/minute. Physical examination revealed patchy lymphoid follicles on the posterior pharyngeal wall and bilateral positive ocular globe compression sign, which were absent at the initial visit. All other meningeal signs, such as nuchal rigidity, jolt accentuation, and Kernig and Brudzinki signs, were negative. Cranial nerves two through twelve, muscle strength, sensation, deep tendon reflexes, gait, and coordination were all intact.

**Table 1:** CSF findings (first and second LP) CSF, cerebrospinal fluid; LP, lumbar puncture

	First LP	Second LP (4 days later)	Normal Range	
Color	clear	clear		
Initial pressure	130	300	70-180	mmH2O
Cell count	2	22	0-2	/μL
Polymorphonuclear cell	2	4		
Lymphocyte		18		
Protein	41	50	15 - 45	mg/dL
Glucose (CSF)	68	65	50-75	mg/dL
Glucose (Blood)	100	100	65-109	mg/dL

Blood tests revealed mild leukocytosis (11,200 cells/μL; normal range, 3,900-9,700 cells/μL) with an elevated neutrophil count (79%) and C-reactive protein level (0.37 mg/dL; normal range, ≤0.3 mg/dL). CSF, obtained by a repeat lumbar puncture, was clear, with an elevated initial pressure (300 mm H2O), cell count of 22 cells/ high power field (monocytes: 18/22, 82%), glucose level of 65 mg/dL, and protein level of 50 mg/dL (Table 1). The initial spinal fluid pressure was somewhat high; however, we believe this may have been attributed to the patient's nervousness at the time of the visit. CSF bacteria smear and culture after 72 hours, India ink test, antimicrobial culture, and polymerase chain reaction (PCR) test for antimicrobials, varicella-zoster virus, and herpes simplex virus (HSV) were negative. After admission, he was treated with ceftriaxone 2 g every 12 hours and acyclovir 400 mg (5 mg/kg) every eight hours. Dexamethasone was not administered because there was no impairment of consciousness, no background of immunosuppression, and a bacterial smear of the spinal fluid was negative. Fundus examination was not performed during this hospitalization due to the absence of ocular conjunctival hyperemia and eye pain. All subsequent culture and spinal fluid PCR tests were negative by day 4 and, therefore, they were all discontinued. The patient’s four-year-old daughter was reported to have gastroenteritis. The patient was diagnosed with aseptic meningitis (probably caused by an enterovirus) based on the family history and his physical and CSF findings. He was treated with intravenous acetaminophen administration for 15 days while hospitalized. The intensity of the pain caused by the compression of the ocular globe decreased on day 10, his fever improved on day 15, and he was discharged on day 19. The patient was also informed to return to our hospital if there was an exacerbation of symptoms. A follow-up check was conducted two weeks after his discharge, and his symptoms did not recur.

## Discussion

In this case, we suspected meningitis based on a positive ocular globe compression sign and made a diagnosis of aseptic meningitis by repeating the lumbar puncture. Furthermore, from the patient’s family history and physical findings of patchy lymphoid follicles on the posterior pharyngeal wall, we considered that viral infection might have been the cause of this case of aseptic meningitis.

In cases of viral meningitis in which a pathogen is identified, the most common cause is non-polio human enteroviruses, accounting for up to 46% of cases, followed by HSV-2 (31%), varicella zoster virus (11%), and HSV-1 (4%) [3]. In contrast, HSV-1 is the most common cause of sporadic encephalitis [4].

Generally, the clinical course of viral meningitis is 7-18 days for enterovirus, five to six days for lymphocytic choriomeningitis virus, 7-10 days for mumps virus, and two to five days for HSV-2 recurrent benign lymphocytic meningitis [5]. In this case, the patient recovered in 16 days and based on the clinical course, the most likely cause of the patient’s symptoms was enteroviral infection. The most frequently reported symptoms of enterovirus meningitis are headache (100%), photophobia (87.5%), nausea (67.5%), vomiting (65%), fever (65%), and neck stiffness (62.5%) [6]; however, in this case, the patient’s only symptoms were headache, fever, and chills.

A previous study reported the sensitivity and specificity (respectively) of meningeal tests as follows: nuchal rigidity (46.1%, 71.3%), jolt accentuation test (52.4%, 71.1%), Kernig’s sign (22.9%, 91.2%), and Brudzinski’s sign (27.5%, 88.8%) [7]. The ocular globe compression sign (applying digital pressure to both eyeballs and observing the presence and degree of pain reaction to the stimulus) was proposed in 2002 [8] and has sensitivity (34.5%), specificity (78.6%), positive (62.5%) and negative (53.7%) predictive values of ocular globe compression sign similar to those of nuchal rigidity. Moreover, the agreement between the two independent observers was fair (Kappa=0.65). The positive and negative likelihood ratio (LR) of this exam is as follows: positive LR, 1.61; negative LR, 0.83 [9]. The mechanism of the ocular globe compression sign can be attributed to the anatomical relationship between the optic nerve and the surrounding subarachnoid space, which may reflect the effects of increased intracranial pressure. Therefore, ocular compression may induce pain in patients with meningitis. Further investigation of the mechanism and its relationship to the clinical stage is expected in the future. The advantage of this test is that it is easy to perform, but it should not be performed on patients with glaucoma. In addition, the procedure should be performed in a short period of time, as the pressure on the eyes may cause bradycardia. In our patient, all the meningeal signs, except for the ocular globe compression sign, were negative. Moreover, with improvement in his symptoms, the ocular globe compression sign became negative. Further studies are needed to clarify the clinical significance of the ocular globe compression sign in the diagnosis of aseptic meningitis and its relationship with the clinical course. In a previous study of patients with suspected meningitis and initial negative CSF findings, 88% had an elevated CSF cell count on repeat lumbar puncture [10]. However, the sensitivity and specificity of repeat CSF tests in viral meningitis remain unknown. PCR testing for viruses is not routinely available; therefore, clinical signs are useful in the diagnosis of aseptic meningitis. No PCR examination was performed on our patient; conducting PCR testing on CSF could have helped diagnose the cause of aseptic meningitis in the sample taken from the first lumbar puncture. However, it would not have changed management. Recently, it was reported that coronavirus disease 2019 (COVID-19) can disrupt the healthcare system and clinical environment and affect diagnosis due to anchoring bias. Thus, it may take longer to diagnose aseptic meningitis correctly, especially during the time of the current COVID-19 pandemic [11]. A diagnosis of meningitis should not be excluded if the first lumbar puncture is normal. It is important to repeat the CSF examination and evaluate the meningeal sign tests to reduce the risk of missing the diagnosis, especially when symptoms worsen or the cause is unclear.

## Conclusions

Aseptic meningitis is diagnosed using the clinical and CSF findings of meningeal inflammation. Meningitis should not be excluded, even if the first lumbar puncture is normal. To reduce the risk of misdiagnosis, repeated lumbar punctures and evaluation of all meningeal signs, including the ocular globe compression sign, are useful.
